# Biological Evaluation and Molecular Docking Study of Ferrociphenol as an Antimelanogenic Agent

**DOI:** 10.1155/bri/5240884

**Published:** 2026-07-26

**Authors:** Emna Ketata, Aissette Baanannou, Wajdi Ayadi, Pascal Pigeon, Aref Neifar, Siden Top, Saber Masmoudi, Gérard Jaouen, Mehdi El Arbi, Ali Gargouri

**Affiliations:** ^1^ Laboratory of Molecular Biotechnology of Eukaryotes, Center of Biotechnology of Sfax, University of Sfax, Sfax, Tunisia, fss.rnu.tn; ^2^ Laboratory of Molecular and Cellular Screening Processes, Center of Biotechnology of Sfax, University of Sfax, Sfax, Tunisia, fss.rnu.tn; ^3^ Chimie ParisTech, PSL University, Paris, France; ^4^ Institut Parisien de Chimie Moléculaire (IPCM), Sorbonne University, CNRS, Paris, France; ^5^ Higher Institute of Biotechnology of Sfax, University of Sfax, Sfax, Tunisia, fss.rnu.tn; ^6^ Laboratory of Enzyme Engineering and Microbiology, National School of Engineering of Sfax, University of Sfax, Sfax, Tunisia, fss.rnu.tn

**Keywords:** docking, ferrociphenol, melanogenesis, melanoma, tyrosinase, zebrafish

## Abstract

Cutaneous hyperpigmentation disorders are associated with abnormal accumulation of melanin pigments, which can be treated using depigmenting agents. In the present study, we investigated the effect of ferrociphenol (Fc‐diOH), an organometallic intermediate used in the synthesis of hydroxy‐ferrocifen derivatives and previously reported as a tyrosinase inhibitor, on melanogenesis in B16F10 melanoma cells. Cell viability, melanin content, and tyrosinase activity assays demonstrated that treatment with Fc‐diOH significantly reduced intracellular melanin levels and tyrosinase activity by 32% and 25%, respectively, in B16F10 cells at 25 nM without inducing significant cytoxicity. Furthermore, the antimelanogenesis activity of Fc‐diOH was confirmed in vivo using zebrafish (*Danio rerio*) embryos. Fc‐diOH treatment at concentrations of 0.5 and 2 μM significantly inhibited melanin production and tyrosinase activity in zebrafish embryos without affecting embryonic development or viability. In addition, molecular docking analysis demonstrates that the *p*‐hydroxyphenyl groups of Fc‐diOH make close contacts with the active site of a predicted human tyrosinase model, compared to arbutin and phenylthiourea. Taken together, these results strongly suggest that Fc‐diOH decreases tyrosinase activity and thereby negatively regulates melanogenesis in both B16F10 cells and zebrafish embryos. Therefore, Fc‐diOH represents a promising candidate as a depigmentation agent for the treatment of hyperpigmentation disorders.

## 1. Introduction

Melanin is a biopolymer pigment synthesized in melanosomes (membrane‐bound organelles) within the melanocytes, via a process called melanogenesis. The type, distribution, and amount of melanin in melanocytes present in the basal layer of the epidermis and in hair follicles determine the skin and hair color of humans and animals [1–3]. Melanogenesis involves both enzymatically catalyzed and spontaneous chemical reactions. Tyrosinase catalyzes the rate‐limiting step in melanogenesis. It catalyzes the hydroxylation of L‐tyrosine to 3,4‐dihydroxy‐phenylalanine (L‐DOPA) and of L‐DOPA to dopaquinone [4]. Following the production of dopaquinone, two main pathways branch off, ultimately resulting in the synthesis of two types of melanin, brown–black eumelanin and yellow–red pheomelanin [5, 6].

In the case of eumelanin synthesis, dopaquinone is converted through a redox exchange into L‐dopachrome and then to mixtures of 5,6‐dihydroxyindole (DHI) and 5,6‐dihydroxyindole‐2‐carboxylic acid (DHICA), according to the decarboxylation rate and the activity of tyrosinase‐related protein 2 (Trp2), respectively. Finally, further oxidation of these dihydroxyindoles by tyrosinase or tyrosinase‐related protein 1 (Trp1) leads to the formation of indolequinones, which polymerize to form eumelanin [7].

Functionally, melanin protects the skin from various types of ionizing radiation, including ultraviolet (UV) radiation, and plays a crucial role in the scavenging of free radicals [8, 9]. However, genetic, hormonal, and environmental factors can lead to an excessive production of melanin in the skin [10–12] and cause diseases including melanoma [8] and pigmentary disorders, such as melasma, age spots, and sites of actinic lesions [13, 14]. Therefore, several melanogenesis inhibitors are currently used as pharmaceutical or cosmetic additives [15, 16]; however, due to the low stability of their formulation and undesirable side effects, their use is still limited [16]. Due to these safety concerns, the ongoing search for potential new molecules preventing pigment disruption continues to progress and attracts increasing interest. Various strategies have been employed to identify melanogenesis inhibitors, including in silico, in vitro, and in vivo approaches [17–20].

Recently, zebrafish (*Danio rerio*) have emerged as a valuable in vivo model for evaluating the depigmenting activity of small molecules [21–24], and for screening compounds that control pigment cell development [25]. In addition to its numerous advantages, including its small size, abundant offspring, and progeny and high efficiency of drug penetration through the skin and gills, the zebrafish embryo exhibits strong physiological and genetic similarity to mammals. More importantly, the effect of the chemical compound on melanogenesis can be assessed by simple observation of the formation of pigmentation on the surface of this animal without any complicated experimental procedures [25, 26]. Like other vertebrates, zebrafish have pigment cells from two distinct embryonic sources. Those of the epidermis and dermis arise from the neural crest, while those that make up the outermost layer of the retina, the retinal pigment epithelium, derive from the optic cupula and begin in the retinal epithelium and in the melanophores. Pigment cells grow rapidly, and within hours, they become an important feature of the embryo [27].

Fc‐diOH, also called ferrociphenol, is an organometallic compound that first served as a simple intermediate for the synthesis of hydroxy‐ferrocifen [28], a compound analogous to hydroxy‐tamoxifen, which is the bioactive form of tamoxifen, a selective estrogen receptor modulator (SERM) [29]. Although Fc‐diOH is not a SERM itself, due to the absence of the dimethylamino‐alkyl chain, it exhibits a strong antiproliferative effect on human breast cancer cell lines MCF‐7 (estrogen receptor positive; ER+) and MDA‐MB‐231 (estrogen receptor negative; ER−) with IC_50_ values of 0.7 and 0.6 μM, respectively [28].

In fact, almost all compounds having the [ferrocenyl‐double bond‐*p*‐phenol] motif have cytotoxic activities on various cell lines, but the ferrocenyl and phenol moieties must be in *trans* configuration. For this reason, having two *p*‐hydroxyphenyl groups, Fc‐diOH still possesses this pharmacophore moiety. Some diphenol derivatives have been improved, such as the cyclic version (ferrocenophane) of Fc‐diOH [28]. All compounds bearing this motif, including the ferrocifen itself, have been classified as belonging to a series called the “Ferrociphenol series.”

In addition to its antiproliferative effects, Fc‐diOH has been reported to possess other biological activities, including antimicrobial activity against several microorganisms such as *Pseudomonas aeruginosa*, *Staphylococcus aureus*, *Candida albicans* [30], *Pseudomonas savastanoi*, *Fusarium solani, and Aspergillus tumefaciens* [31]. In addition, we recently reported that among 20 compounds tested against Sepia tyrosinase, a key enzyme in melanogenesis, Fc‐diOH was the most potent inhibitor, acting through a competitive inhibition mechanism [32]. Structural comparisons with related derivatives revealed that the presence of a hydroxyl radical on the phenyl moieties makes it more active; this is due to the structural homology between the substrate (L‐tyrosine) and its hydroxyphenyl group [32]. Therefore, we hypothesized that Fc‐diOH represents a key pharmacophore with strong antimelanogenesis potential. The aim of the present study was to investigate the depigmenting effect of Fc‐diOH in vitro using the murine melanoma cell line B16F10 and in vivo using a zebrafish model. In addition, molecular docking analysis was performed to support the interaction between Fc‐diOH and tyrosinase.

## 2. Materials and Methods

### 2.1. Reagents

**FIGURE 1:**
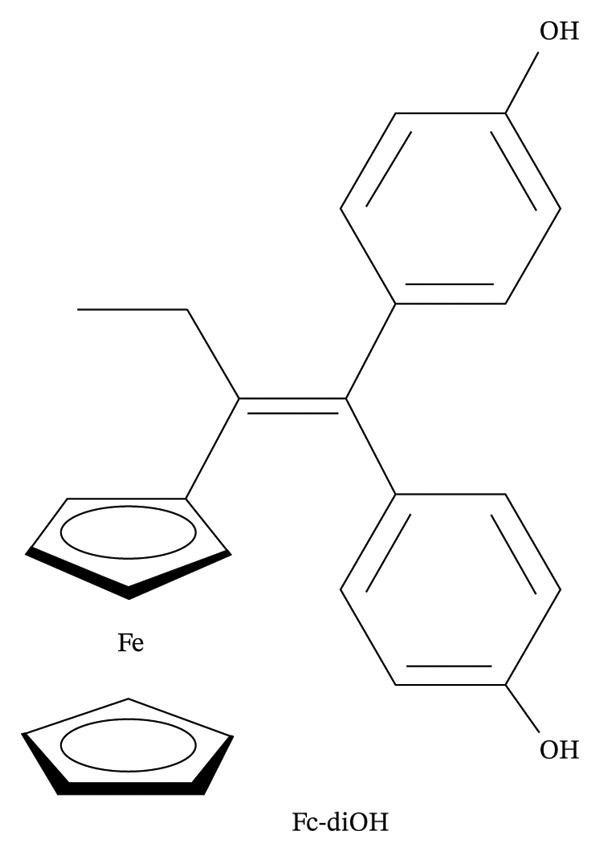
Structure of Fc‐diOH.

L‐DOPA, gentamicin, and MTT (thiazolyl blue tetrazolium bromide) were purchased from BioBasic, Canada. IBMX, arbutin, and melanin were obtained from Sigma‐Aldrich, Germany. RPMI‐1640 medium, fetal bovine serum, and trypsin‐EDTA (0.05%) were purchased from Gibco Life Technologies, Paisley, UK. Fc‐diOH (Figure 1) was synthesized as previously described [28].

### 2.2. Murine Melanoma Cell Line Culture

The B16F10 melanoma cell line was maintained in RPMI 1640 medium supplemented with 10% fetal bovine serum and gentamicin (50 μg/mL). The cells were cultured at 37°C in a humidified atmosphere containing 5% CO_2_.

### 2.3. MTT Assay

Cell viability was determined using the MTT assay as previously described [33]. B16F10 cells were seeded at a density of 10^4^ cells/well in a 96‐well tissue culture plate. After 24 h of incubation, cells were treated with different concentrations of Fc‐diOH for an additional 24 h.

Following treatment, the culture medium was removed and replaced with fresh medium containing MTT solution (1 mg/mL), and the plates were incubated for 4 h at 37°C. Subsequently, the formed formazan crystals were dissolved in SDS (10%) and further incubated overnight at room temperature. Absorbance was measured at 570 nm using a microplate reader (Thermo Scientific Varioskan Lux). Data were representative of three independent experiments.

### 2.4. Determination of Melanin Content

Melanin content was assessed using a previously described method with slight modifications [34]. Briefly, B16F10 cells were seeded at a density of 2 × 10^5^ cells per well in a 6‐well plate. After 24 h of incubation, cells were incubated without or with Fc‐diOH at concentrations ranging from 5 to 25 nM in the presence of IBMX (3‐isobutyl‐1‐methylxanthine) at 100 μM to induce melanogenesis. After 48 h of incubation, cells were harvested and washed twice with PBS. The pelleted cells were dissolved in 1 N NaOH for 1 h at 60°C. The mixture was then vortexed vigorously to solubilize the melanin pigment. Absorbance was measured at 410 nm, and the melanin content was quantified using a synthetic melanin standard.

### 2.5. Measurements of Intracellular Tyrosinase Activity

Intracellular tyrosinase activity of B16F10 cells was determined by measuring the oxidation rate of the L‐DOPA substrate, as previously described [34]. Cells were seeded at a density of 2 × 10^5^ cells per well in a 6‐well plate. After 24 h of incubation, cells were treated with different concentrations of Fc‐diOH, in the presence or absence of IBMX (100 μM) for an additional 24 h.

Cells were then harvested, washed with PBS, frozen at −20°C, and thawed in 200 μL of lysis buffer (10 mM sodium phosphate pH 6.8, 1% Triton X‐100, and 1 mM PMSF).

Cell lysates were centrifuged at 13,000 rpm for 20 min at 4°C, and the resulting supernatant was used to determine crude tyrosinase activity. Protein concentration was determined by the Bradford assay with bovine serum albumin as a standard.

For the enzyme assay, 50 μg of total protein was added to each well of a 96‐well plate. The enzyme assay was initiated by the addition of L‐DOPA and sodium phosphate buffer (pH 6.8), and absorbance at 475 nm was measured every 10 min for at least 1 h at 37°C using a colorimetric microplate reader. Tyrosinase activity was calculated and expressed relative to the IBMX‐induced control.

### 2.6. Acute Toxicity Test on Zebrafish Embryos

Adult zebrafish were maintained in aquaria with an alternating 14/10 h light/dark cycle at 28°C. Zebrafish husbandry and experimental procedures were performed according to international guidelines on the protection of experimental animals. Because embryos used in this work were no more than 5 days old, no license was required by the Council of Europe (Directive 2010/63/EU) or the local authority. Synchronized embryos were obtained from natural spawning induced in the morning by light onset. Collected zebrafish embryos were distributed in 24‐well plates (20 embryos per well) containing 2 mL of E3 medium and treated with Fc‐diOH from 4 h to 48 hpf (hours postfertilization) with various concentrations ranging from 0 to 10 μM. The observation of sublethal and lethal morphological parameters (embryonic coagulation, absence of somite formation, nondetachment of the tail bud, and absence of heart rate) was carried out at 1 and 2 dpf using the Stemi 2000‐C stereoscope (Carl Zeiss, Germany) equipped with an AxioCam 105 Colors CCD camera (Carl Zeiss).

### 2.7. Evaluation of Depigmenting Activity in Zebrafish Embryos

The depigmentation effect in the zebrafish model was evaluated according to the method described by Choi et al. [21]. Thirty zebrafish embryos were treated with Fc‐diOH at various concentrations (0.1–2 μM) from 9 to 48 hpf. Embryos treated with 50 μM *N*‐phenylthiourea (PTU) served as the positive control. Changes in zebrafish pigmentation were observed under a stereomicroscope (Zeiss Stemi 2000‐C), and images were photographed under a digital camera (Zeiss Axiocam 105 color). Each group of embryos was sonicated for 5 min at 70% amplitude in ice‐cold lysis buffer containing 10 mM sodium phosphate pH 6.8, 1% Triton X‐100, and 1 mM phenylmethylsulfonyl fluoride (PMSF). The lysate was centrifuged at 12,000 rpm for 20 min at 4°C. The pellet was suspended in 100 μL of NaOH (1 N) and incubated at 100°C for 1 h. The samples were then vortexed vigorously to solubilize the melanin pigment. The optical density of the supernatant was measured at 410 nm using a colorimetric microplate reader (Thermo Scientific Varioskan Lux).

For the determination of tyrosinase activity, 250 μg of total protein from each lysate was added to a reaction mixture containing 50 mM phosphate buffer (pH 6.8) and 2.5 mM L‐DOPA. After incubation at 37°C for 60 min, dopachrome formation was quantified by measuring absorbance at 475 nm. Tyrosinase activity and melanin content were expressed as a percentage relative to the control group, which was defined as 100%.

### 2.8. Homology Modeling and Docking Studies on Human Tyrosinase (hTYR)

Because the 3D crystal structure of hTYR is not currently available, homology modeling was performed to predict the 3D structure of hTYR. The amino acid sequence of hTYR (529 AA, ID: P14679) was retrieved from the UniProt Knowledge database (https://www.uniprot.org/uniprot/P14679).

Tyrosinase from *Bacillus megaterium* (pdb: 3ntm), which shares 32.8% sequence identity with the target protein sequence, was selected as the template for model construction. Homology modeling was carried out using the SWISS‐MODEL server (https://swissmodel.eexpasy.org).

Molecular docking studies were subsequently performed to predict the interactions between hTYR and Fc‐diOH, arbutin, and N‐PTU using Autodock/Vina [35]. The docking grid was centered on the predicted active site of tyrosinase. The docking grid box dimensions were set to 30 × 30 × 40 grid points, with grid center coordinates of 11, −11, and 6 along the x‐, y‐, and *z*‐axes, respectively. The best conformations with the lowest binding free energy were selected as the most favorable conformations and further analyzed using Discovery Studio 2017 R2 Client.

### 2.9. Statistical Analysis

Differences between the results of the melanin content assay and the intracellular tyrosinase activity assay were assessed for statistical significance using Student′s *t*‐test using SPSS 13.0 statistical software (SPSS). Differences were considered statistically significant at ^∗^ *p* < 0.05 and ^∗∗^ *p* < 0.01.

## 3. Results

### 3.1. Inhibition of Melanogenesis by Fc‐diOH in B16F10 Cells

**FIGURE 2:**
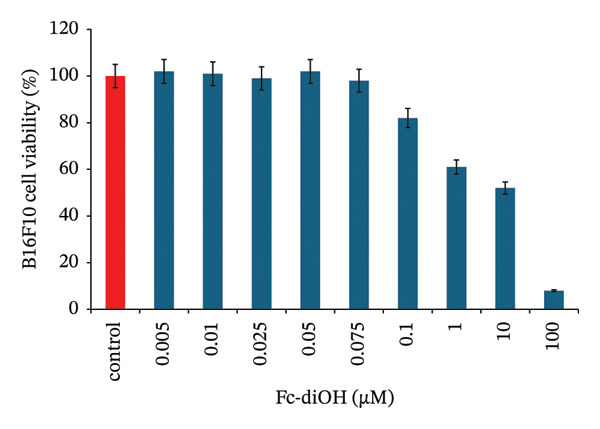
Effect of Fc‐diOH on B16F10 cell viability at various concentrations ranging from 0.005 to 100 μM.

The effect of Fc‐diOH (Figure 1) on cell viability was evaluated using the MTT assay. The results showed that it had no significant cytotoxic effect on B16F10 cell lines at concentrations ranging from 5 to 75 nM (Figure 2). In contrast, a marked reduction in cell viability was observed at a concentration of 100 nM. This cytotoxic effect on B16F10 melanoma cells is consistent with previously reported findings by Bruyère et al. [36].

**FIGURE 3:**
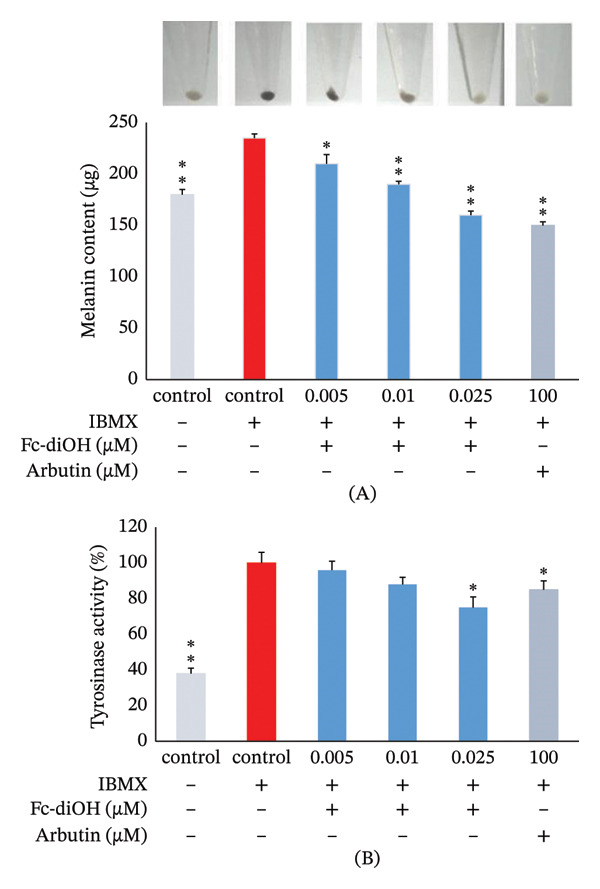
Effect of Fc‐diOH on melanin production in B16F10 cells treated with 0.005, 0.01, and 0.025 μM Fc‐diOH or 100 μM arbutin. Representative images of cell pellet colors corresponding to the different melanin measurements are shown above the diagram (A). (B) Intracellular tyrosinase activity in B16F10 cells treated with 0.005, 0.01, and 0.025 μM Fc‐diOH or 100 μM arbutin. Data are presented as the mean ± S.D. of three independent experiments. Values are significantly different compared with the IBMX‐treated control. ^∗^ *p* < 0.05 and ^∗∗^ *p* < 0.01.

To investigate the effects of Fc‐diOH on melanogenesis, intracellular melanin content was measured in B16F10 cells following induction of melanogenesis with IBMX. Cells were subsequently treated with noncytotoxic concentrations of Fc‐diOH (5, 10, and 25 nm), with arbutin used as a positive control. Fc‐diOH showed a significant inhibitory effect on melanin production in a dose‐dependent manner (Figure 3A). Treatment with 25 nM Fc‐diOH resulted in a 32% reduction in melanin content, whereas arbutin, applied at a substantially higher concentration, produced a slightly stronger inhibitory effect, reducing melanin production by 36% in B16F10 melanoma cells. Representative images of the cell pellets included in Figure 3A showed pigmentation changes consistent with the quantitative melanin measurements (Figure 3A), where darker pellets indicated higher melanin content and lighter pellets reflected reduced melanogenesis after Fc‐diOH treatment.

### 3.2. Tyrosinase Inhibition in B16F10 Cells by Fc‐diOH

To assess the effect of Fc‐diOH on tyrosinase activity and to elucidate the mechanism underlying the inhibition of melanogenesis in IBMX‐stimulated B16F10 melanoma cells, intracellular tyrosinase activity was measured in cells treated at concentrations of 5, 10, and 25 nM. Arbutin (100 μM) was used as a positive control. The results showed that treatment with 25 nM Fc‐diOH reduced intracellular tyrosinase activity by approximately 25%, whereas treatment with 100 μM arbutin resulted in a 15% reduction (Figure 3B). Fc‐diOH reduced intracellular tyrosinase activity more effectively than arbutin, despite being used at a substantially lower concentration.

The decrease in tyrosinase activity obtained at 25 nM Fc‐diOH correlates with the decrease in melanin content described above at the same concentration.

### 3.3. Evaluation of the in vivo Depigmenting Effect of Fc‐diOH on Zebrafish Embryos

**FIGURE 4:**
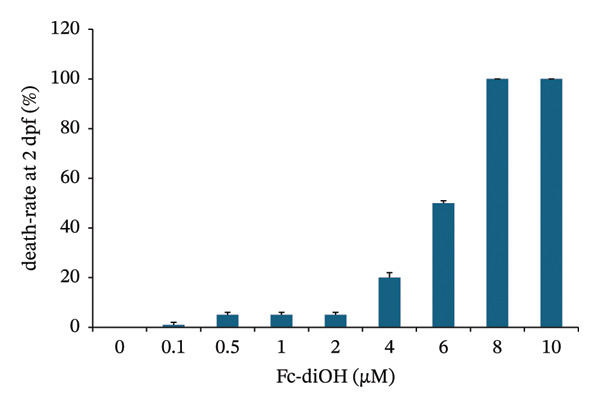
Effects of Fc‐diOH on the death rate of zebrafish embryos after 2 days postfertilization (dpf) at various concentrations ranging from 0.1 to 10 μM.

The zebrafish model was used as an in vivo system to evaluate the inhibitory effect of Fc‐diOH on melanogenesis (Figure 4). 1‐phenyl‐2‐thiourea was used as a positive control to inhibit melanin production in zebrafish, as previously described [27] by blocking all steps of tyrosinase‐dependent melanin synthesis [19].

In order to identify the toxic concentration of Fc‐diOH in zebrafish embryos, an acute toxicity assay was performed from 2 h postfertilization (hpf) to 2 days postfertilization (dpf) (Figure 4). Treatment with Fc‐diOH at concentrations of 1 and 2 μM had no effect on embryo development or viability. In contrast, exposure to higher concentrations (4, 6, and 8 μM) resulted in dose‐dependent lethality rates of 20%, 54%, and 100%, respectively.

**FIGURE 5:**
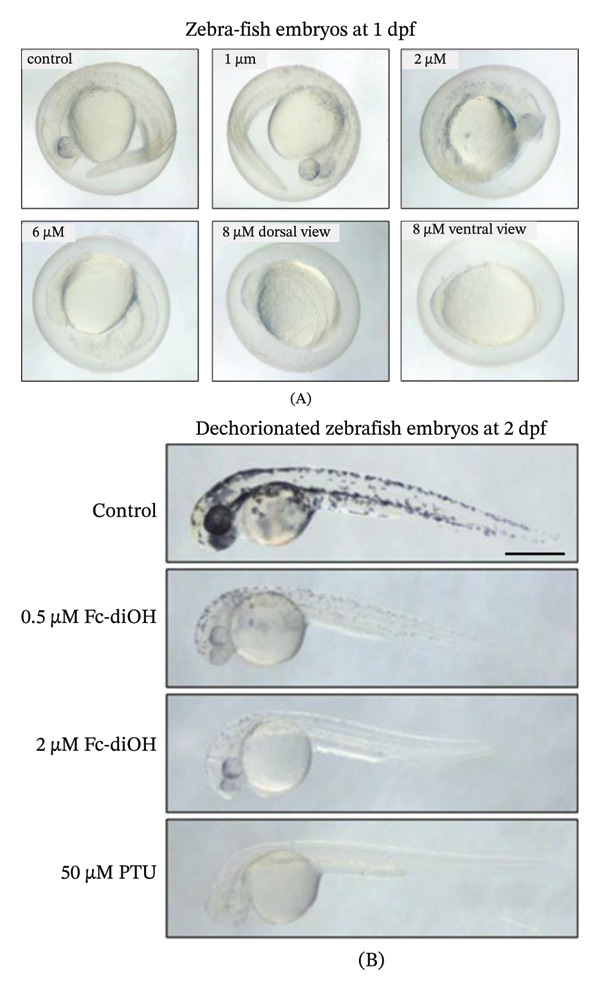
Effects of Fc‐diOH on melanogenesis in zebrafish embryos. The effects on the pigmentation of zebrafish embryos were observed by microscopy. (A) Zebrafish embryos at 1 day postfertilization (dpf). Untreated embryo: control, treated embryos with 1, 2, 6, and 8 μM of Fc‐diOH. (B) Dechorionated zebrafish embryos at 2 dpf. Untreated embryo: control, treated embryos with 0.5 and 2 μM of Fc‐diOH, and embryos treated with 50 μM of 1‐phenyl‐2‐thiourea (PTU).

**FIGURE 6:**
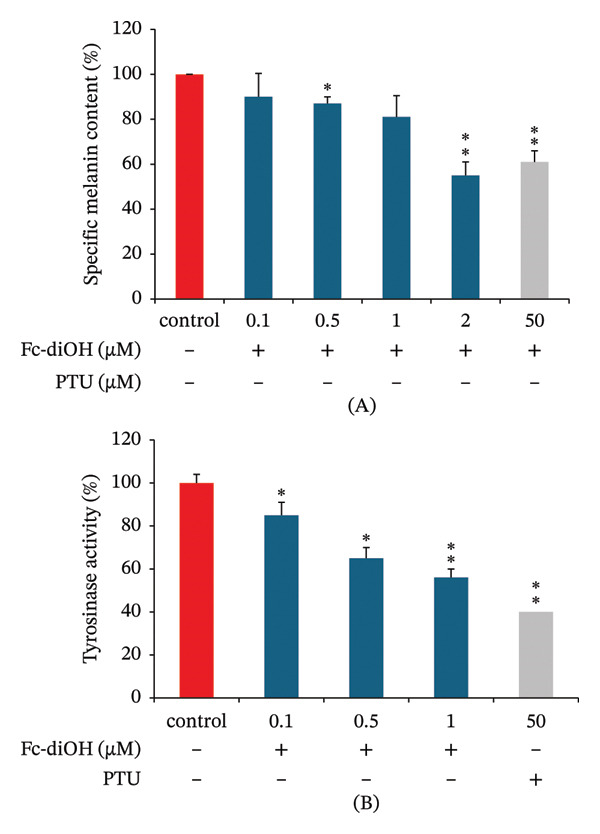
Inhibitory effect of Fc‐diOH on melanin production in zebrafish embryos at concentrations ranging from 0.1 to 2 μM Fc‐diOH and 50 μM PTU. (A) Percentage of intracellular tyrosinase activity at concentrations ranging from 0.1 to 1 μM Fc‐diOH and 50 μM PTU. (B) Data are presented as the mean ± S.D. of three independent experiments. Values are significantly different compared with the control. ^∗^ *p* < 0.05 and ^∗∗^ *p* < 0.01.

As shown in Figure 5A, zebrafish embryos at 1‐day postfertilization (dpf) treated with Fc‐diOH had normal development at concentrations of 1 and 2 μM, whereas development delay was observed at concentrations of 6 and 8 μM. Interestingly, treatment with Fc‐diOH at 0.5 and 2 μM led to remarkable depigmentation of zebrafish embryos (Figure 5B). To further assess the inhibitory activity of Fc‐diOH, tyrosinase activity and total melanin content were quantified in whole‐embryo extracts. Fc‐diOH showed a dose‐dependent inhibitory effect on both melanin production (Figure 6A) and tyrosinase activity (Figure 6B). Importantly, Fc‐diOH induced a depigmenting effect at a concentration as low as 0.1 μM, which is substantially lower than the effective concentration of PTU required to achieve comparable depigmentation.

### 3.4. Molecular Docking Analysis

**FIGURE 7:**
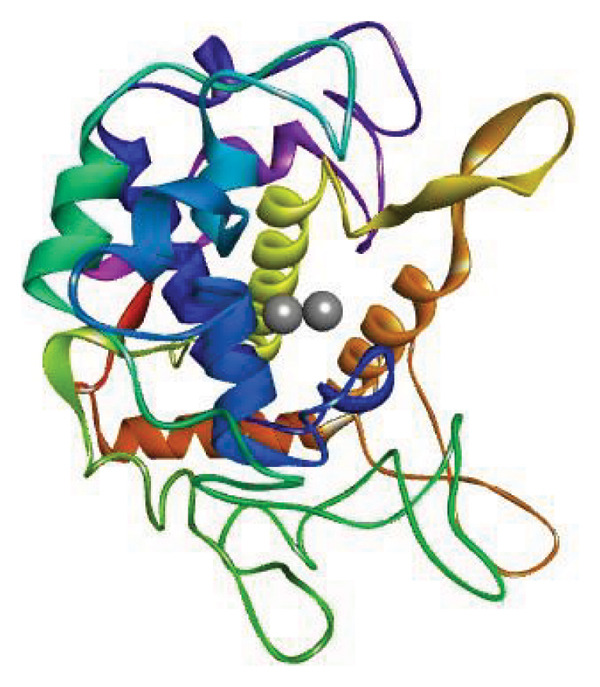
Predicted human tyrosinase model.

Molecular docking analysis was applied to investigate the binding interactions of Fc‐diOH, arbutin, and PTU with the predicted 3D structure of hTYR using AutoDock Vina, with the aim of elucidating possible predicted interactions. The 3D crystal structure of hTYR was generated by homology modeling using the SWISS‐MODEL server, with tyrosinase from *Bacillus megaterium* (bmTYR; PDB ID: 3ntm) as a template (Figure 7).

**FIGURE 8:**
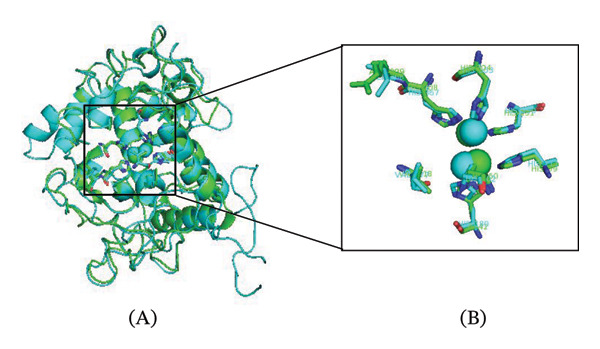
(A) Superposition of the hTYR homology model (blue sticks) on bmTYR (green sticks). (B) Close view of hTYR and bmTYR active site, the catalytic residues of bmTYR are (His 42, His 60, His 69, His 204, His 208, His 231, Val 2018, and Arg 209) and catalytic residues of hTYR predicted model are (His 180, His 202, His 211, His 363 His 367, His 390, Val 377, and Ile 368).

The superposition of the hTYR model on bmTYR revealed a conserved catalytic cavity with substitution of the catalytic residues of bmTYR His42, His60, His69, His204, His208, His231, Val201, and Arg209 [37] corresponding to predicted catalytic residues of hTYR model His180, His202, His211, His363 His367, His390, Val377, and Ile368, respectively (Figure 8). The validated hTYR model was subsequently used to perform the molecular docking studies of Fc‐diOH, arbutin, and PTU.

**FIGURE 9:**
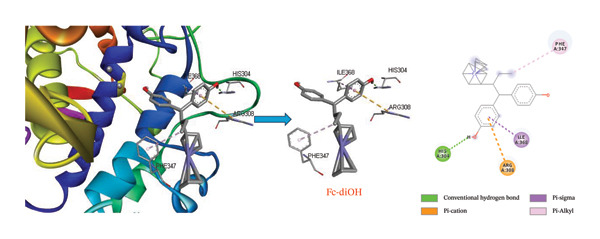
Representation of the putative binding modes of Fc‐diOH in the “pocket” of the human tyrosinase model. The green dotted lines indicate H‐bond interactions, the pink and purple dotted lines indicate hydrophobic interactions, the orange dotted lines indicate electrostatic interactions, and the two gray spheres indicate copper ions at the active site.

Docking results indicated that the *p*‐hydroxyphenyl group of Fc‐diOH could interact with the catalytic residue Ile 368 of hTYR, which corresponds to the catalytic residue Arg209 in bmTYR, a residue known to play a role in substrate binding orientation based on its flexibility and spatial positioning [37]. Additional interactions with His304 and Arg308 were also observed (Figure 9 and Table 1). These findings are consistent with our previous results and further support the competitive inhibition mechanism of Fc‐diOH against tyrosinase activity [32].

**TABLE 1:** Binding energy and interacting residues of human tyrosinase with inhibitors from AutoDock‐Vina.

Compounds	Interactions with human tyrosinase model	
	Binding energy (KJ/mol)	Interacting residues
Fc‐diOH	−8.1	H‐bonds: His 304
		Hydrophobic interactions: Ile 368, Phe 347
		Electrostatic interactions: Arg 308
Arbutin	−6.4	H‐bonds: Glu 203, Ser 360
		Hydrophobic interactions: His 202, His 367, Val 377
PTU	−4.5	H‐bonds: Gln 286
		Hydrophobic interactions: Gln 376
		Pi–sulfur interactions: His 285, Cys 289

**FIGURE 10:**
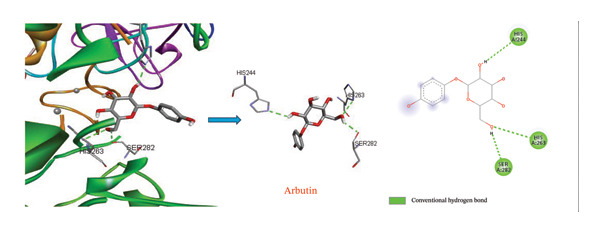
Representation of the putative binding modes of arbutin in the “pocket” of the human tyrosinase model. The green dotted lines indicate H‐bond interactions, and the two gray spheres indicate copper ions at the active site.

In contrast, the hydroxyphenyl group of arbutin was predicted to interact with the catalytic residues His202, His367, and Val377 of hTYR via hydrophobic interactions (Figure 10 and Table 1).

**FIGURE 11:**
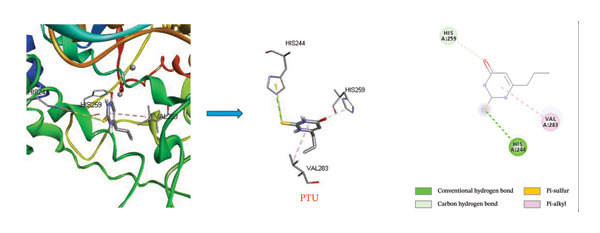
Representation of the putative binding modes of PTU in the “pocket” of the human tyrosinase model. The green dotted lines indicate H‐bond interactions, the pink dotted lines indicate hydrophobic interactions, the yellow dotted lines indicate pi–sulfur interactions, and the two gray spheres indicate copper ions at the active site.

PTU was found to interact with residues surrounding the hTYR active site, including Gln 286, Gln 376, His 285, and Cys 289 (Figure 11 and Table 1). Among the tested ligands, Fc‐diOH exhibited the lowest binding free energy (−8.1 kcal/mol), indicating the strongest predicted interaction with hTYR (Table 1).

## 4. Discussion

Melanogenesis is responsible for skin color and plays an important role in protecting the skin from sun‐related damage. However, excessive melanin production can adversely affect appearance and is associated with various hyperpigmentation disorders. Therefore, tyrosinase inhibitors are widely used for the treatment of skin hyperpigmentation and as skin whitening agents [38]. We have previously demonstrated that the Fc‐diOH acts as a competitive inhibitor of Sepia tyrosinase activity [32]. Nevertheless, further investigations using biological models more closely related to humans are required to better elucidate its effects on melanogenesis.

Here, we examined the antimelanogenic potential of Fc‐diOH using both in vitro and in vivo models, namely B16F10 murine melanoma cells and zebrafish embryos. In addition, molecular docking analyses were conducted to assess whether Fc‐diOH can directly bind to tyrosinase with high affinity and thereby inhibit its activity.

Inhibition of melanogenesis in B16F10 melanoma cells was associated with a significant reduction in intracellular melanin and tyrosinase activity at concentrations that did not affect cell viability. Notably, Fc‐diOH reduced intracellular tyrosinase activity by 32% at 25 nM, more potently than arbutin (15% at 100 μM) (Figure 3B).

In the zebrafish model, treatment with 6 μM Fc‐diOH resulted in approximately 50% embryo mortality after 2 days postfertilization (Figure 4), a concentration that is very close to the reported LC_50_ of tamoxifen in zebrafish (8 μM) [39]. Importantly, Fc‐diOH induced a depigmenting effect at a concentration of 0.1 μM without affecting embryonic development. This effective concentration is approximately 20‐fold higher than that required in B16F10 cells, which may be explained by differences in compound uptake, metabolism, or bioavailability within the developing embryo.

The observed reduction in melanin content in both zebrafish embryos and B16F10 cells treated with Fc‐diOH may result from direct inhibition of tyrosinase activity and/or downregulation of tyrosinase expression and other melanogenesis enzymes. Further molecular studies will be required to fully elucidate these mechanisms.

Consistent with the experimental results obtained in both in vivo and in vitro models, as well as with our previous findings, Fc‐diOH exhibited a competitive inhibition pattern against Sepia tyrosinase activity [32]. Furthermore, to confirm the inhibitory effect of Fc‐diOH on tyrosinase activity, molecular docking analyses were performed using the predicted model (hTYR) structure as the target molecule. Docking results revealed that the *p*‐hydroxyphenyl groups of Fc‐diOH interact with key residues within the active site of hTYR, including His304, Arg308, and Ile368 (Figure 9 and Table 1). The residue Ile 368 of the active site of hTYR corresponds to the Arg209 in *Bacillus megaterium* tyrosinase (Figure 8), which plays a role in the binding orientation of the substrate depending on its flexibility and position [37]. These results suggest that Fc‐diOH establishes close contacts with the active site of tyrosinase, which could be due to structural similarities between its *p*‐hydroxyphenyl moieties and the natural substrate L‐tyrosine. Supporting these observations, a previous docking study using *Agaricus bisporus* tyrosinase and Molegro Virtual Docker indicated that the phenolic groups of Fc‐diOH form hydrogen bonds with His244, Thr84, and Asn320 within the enzyme’s binding pocket [39]. Arbutin, a hydroquinone glucoside compound widely used in pharmaceuticals and cosmetics for its ability to prevent melanin overproduction [40, 41], was predicted to interact with different catalytic residues of hTYR, namely His202, His367, and Val377. This difference in binding patterns may explain the distinct inhibitory mechanisms and relative potencies of Fc‐diOH and arbutin in melanogenesis inhibition.

Additionally, the standard for the depigmenting agent PTU was found to interact with residues surrounding the hTYR active site, including Gln 286, Gln 376, His 285, and Cys 289, with a comparatively weaker binding energy (−4.5 kcal/mol) (Figure 11 and Table 1).

Among the tested compounds, Fc‐diOH exhibited the most favorable binding free energy (−8.1 kcal/mol) and the highest number of interactions with active‐site residues, supporting its stronger predicted inhibitory effect on tyrosinase.

Beyond its antimelanogenic activity, Fc‐diOH has previously been reported to exert antiproliferative effects on hormone‐dependent and hormone‐independent breast cancer cells [9, 28], leukemia cells [42], and B16F10 melanoma cells [36]. Importantly, Fc‐diOH has also been shown to exhibit low toxicity toward healthy cells [42], further supporting its potential therapeutic and cosmetic applications.

In conclusion, this study describes for the first time the inhibitory effect of Fc‐diOH on melanogenesis in B16F10 cells and in the zebrafish embryo, through the reduction of melanin synthesis and intracellular tyrosinase activity. Furthermore, molecular docking analysis revealed that the *p*‐hydroxyphenyl groups of Fc‐diOH establish close interactions with the active site of tyrosinase via hydrogen bonds and hydrophobic interactions. This explains the significant inhibitory effect of Fc‐diOH on melanogenesis and highlights its potential interest for a topical formulation in the cosmetic industry, particularly for the treatment of various hyperpigmentation disorders.

## Author Contributions

**Emna Ketata:** writing–original draft, methodology, investigation, formal analysis, visualization, conceptualization, and data curation. **Aissette Baanannou and Pascal Pigeon**: methodology, writing–review and editing, and validation. **Wajdi Ayadi, Aref Neifar, Siden Top, Saber Masmoudi, Mehdi El Arbi, and Gérard Jaouen**: methodology and writing–review and editing. **Ali Gargouri:** writing–review and editing, supervision, methodology, investigation, conceptualization, validation, project administration, and funding acquisition.

## Funding

No funding was received for this manuscript.

## Disclosure

A previous version of this manuscript was posted on *bioRxiv* (https://www.biorxiv.org/content/10.1101/2025.06.20.660698v1.full).

## Ethics Statement

The care and use of animals were in accordance with the international guidelines on the protection of experimental animals. Because embryos used in this work were no more than 5 days old, no license was required by the Council of Europe (Directive 2010/63/EU) or the local authority.

## Conflicts of Interest

The authors declare no conflicts of interest.

## Data Availability

The data that support the findings of this study are available from the corresponding author upon reasonable request.
